# Active TB infection and its associated factors among HIV-1 infected patients at Jimma medical center, Southwest Ethiopia

**DOI:** 10.1186/s40780-021-00228-5

**Published:** 2021-12-06

**Authors:** Temesgen Mulugeta, Alazar Takale, Belachew Umeta, Behailu Terefe

**Affiliations:** 1 Department of Clinical Pharmacy, School of Pharmacy, Jimma University, Jimma, Oromia Ethiopia; 2 Department of Pharmaceutical Sciences, School of Pharmacy, Jimma University, Jimma, Oromia Ethiopia

**Keywords:** HIV, Active TB, Prevalence, Associated factors, Jimma

## Abstract

**Background:**

Human immune deficiency virus (HIV) increases the susceptibility to primary infection or reinfection and the risk of tuberculosis (TB) reactivation for patients with latent TB. There was no current report on the rate of active TB infection among HIV-1 infected patients in our teaching and referral hospital. Therefore, this study was aimed to determine the prevalence and factors associated with active TB infection among HIV-1 infected patients.

**Methods:**

Hospital-based retrospective cross-sectional study was conducted at the Anti-Retroviral Therapy (ART) chronic follow-up clinic. Systematic random sampling was used to include the patients. A structured questionnaire was used to collect data. Data were analyzed using SPSS version 25. Descriptive statistics were used to describe the findings and multivariate logistic regression was performed to identify factors associated with active TB infection.

**Result:**

150 HIV-1 infected patients (female 54.7%) were included. The median (interquartile range, IQR) age of the patients was 33.5 (25.7, 40.0) years. Twenty-six (17.3%) of the patients had developed active TB infection, which was independently associated with the WHO clinical stage III and IV (AOR: 9.67, 95% confidence interval (CI); 2.21–42.37), *p* = 0.003). The use of isoniazid preventive therapy (IPT) (AOR: 0.123, 95CI; 0.034–0.44, *p* = 0.001) and having good adherence to ART medications (AOR: 0.076, 95CI; 0.007–0.80, *p* = 0.032) was associated with the reduced risk of active TB infection among HIV-1 infected patients.

**Conclusions:**

Advanced WHO clinical stages increased the risk of active TB infection, while the use of IPT and good adherence to ART medications reduced the risk of active TB infection. Therefore, patients with advanced WHO clinical stage should be screened for TB infection, and starting IPT for the candidate patients should be strengthened to reduce the burden of active TB incidence. ART medication adherence should also be supported.

## Introduction

In 2015, there were an estimated 10.4 million cases of Tuberculosis (TB) disease globally, including 1.2 million [11%] among people living with the Human immune-deficiency virus (HIV). Almost 60% of TB cases among people living with HIV (PLWH) were not diagnosed or treated, resulting in 390,000 TB-related deaths. Sub-Saharan Africa bears the brunt of the dual epidemic, accounting for approximately 84% of all deaths from HIV-associated TB in 2018 [1].

HIV infection is the most important risk factor for developing active tuberculosis, which increases the susceptibility to primary infection or reinfection and also the risk of tuberculosis (TB) reactivation for patients with latent TB. It also speeds up the progression of HIV infection to Acquired immunodeficiency syndrome (AIDS) [2]. Untreated latent TB infection can quickly progress to active TB infection in PLWH since the immune system is already weakened [3]. In HIV-infected patients, there is a 5 to 15% annual risk of developing active TB infection [4]. The risk of developing TB is estimated to be between 16 and 27 times greater in PLWH than among those without HIV infection. In order to reduce the incidence of TB infection in PLWH, World Health Organization (WHO) recommends the three I’s strategy: intensified case-finding (ICF), isoniazid preventive therapy (IPT), and infection control (IC) at all clinical encounters. PLWH who are unlikely to have active TB should receive at least 6 months of IPT as part of a comprehensive package of HIV care. However, fewer than 25% of persons living with HIV and who are in care are receiving it [1].

Ethiopia has notified 125,836 new TB cases and enrolled 702 drug-resistant TB cases in 2016. HIV co-infection impedes the TB control efforts contributing to around 8% of annually notified TB cases [5]. Ethiopia has adopted the 6 months-isoniazid (INH) monotherapy for PLWH regardless of their ages and TB-exposed under-five children [5]. Studies from different regions of Ethiopia have reported a higher prevalence of TB infection among HIV-infected patients [6–12]. The factors responsible to increase the risk of TB infection were lower baseline CD4 count <200cell/μL [7–9, 11], WHO clinical stage III and IV [6–8, 10], did not take IPT [7], ambulatory functional status [9, 10], cigarette smoking and hemoglobin below 10 g/dL [6].

However, most of the related published studies were from the Amhara [9, 11–16] and Southern Nations, Nationalities, and People’s (SNNPs) regions [6–8, 10] of the country. There was a scarcity of evidence on the rate of active TB infection among PLWH in our teaching and referral hospital. A study published from our hospital by Gudina et al. [17] was too early and the data may not be reflective of the current status in our setting. Understanding the predictors of active TB infection among HIV-infected patients is important to improve TB/HIV co-infected patients’ management. The findings from this study may help contribute to evidence for the policymakers for the TB control programs and inform clinicians in the areas of TB control and prevention. Therefore, this study was designed to determine and update the prevalence of active TB infection and the associated factors among HIV-1 infected patients at Jimma Medical Center (JMC).

## Materials and methods

### Study area and period

The study was conducted at JMC, the ART chronic follow-up clinic. The study area, Jimma town, Oromia Region, is found 352 km away from the country’s capital city, Addis Ababa in the Southwest direction. The data were collected between January 11 and February 1, 2021

#### Study design

Hospital based retrospective cross-sectional study.

### Study population

The study population is adult HIV-1 infected patients who were registered and on follow-up between September 1, 2017, and September 1, 2020. The patients who developed active TB before HIV infection is confirmed, patients’ cards with incomplete data, and those aged less than 15 years were excluded.

### Sample size

Due to the financial and time feasibility, the sample size was calculated using a single population proportion formula. From the early published study, 16% of the HIV-infected patients had active TB at the time of HIV diagnosis or developed it since then [17]. Assuming a 95% confidence level, 5% degree of freedom (d), the minimum sample size (n0) was calculated using a formula; n = (Zα/2)^2^ p(1-p)/d^2^ = 206. Between September 1, 2017, and September 1, 2020, the number of registered adult HIV-1 infected patients on follow-up was 403. The reduced formula (nf: n/ (1 + n/N)) was used to determine the final sample size, which was 136. For the incomplete data, 10% of the final sample size was added. Thus, the final sample size was 150.

### Sampling techniques

A systematic random sampling technique with a lottery method was adopted for selecting a representative sample. Every two patients’ card was selected to include the participants.

### Data collection procedure

A structured questionnaire was developed after reviewing different literature. Sociodemographic, clinical, and laboratory data were collected. The data were collected by a principal investigator and two other trained Clinical Nurses.

### Data quality assurance

Before the actual data collection, a pretest was done on 10% of the final sample size of patients. Modifications were made to the data collection tool. During the actual data collection, every filled questionnaire was checked for completeness.

### Data processing and analysis

The collected data rechecked for completeness, cleaned and coded. Then, data were entered SPSS version 25 for analysis. Descriptive statistics were performed, and the findings were presented in frequency, percentage, mean (standard deviation, SD), and median (interquartile range, IQR). The tables and pie chart were also used to present the findings. Chi-square was performed to assess the relationship of categorical variables with the outcome. Univariate logistic regression was performed with each explanatory variable and a variable with a *p*-value of 0.25 was retained and fitted into the multivariate logistic regression using the backward method to determine the factors associated with the development of active TB infection. The effect size was reported using the odds ratio with their 95% confidence interval. And the *p*-value < 0.05 was used to declare the significant association.

### Study variables

**Dependent variable**: the presence of active TB infection among HIV-1 infected patients.

**Independent variables***:* patient sociodemographic (age, sex, education status, residence, ethnicity, etc.), baseline disease clinical characteristics, and laboratory data.

### Operational definition

**Tuberculosis (TB):** is an infectious disease caused by the bacterium *Mycobacterium tuberculosis*.

**Active tuberculosis**: refers to symptomatic TB that occurs in patients infected with HIV that is characterized by cough with sputum and blood, chest pains, weakness, weight loss, fever, and night sweats.

**Human immunodeficiency virus (HIV):** is a virus that attacks the immune system, the body’s natural defense system and making a person more vulnerable to infections, and also the virus causes acquired immune deficiency syndrome (AIDS).

**Drug adherence**: in this study, it was reported as good adherence when > 95% of the total dosage units issued were received by the patients, and poor when < 95% of the issued dosage units was received [18].

**The functional status** of the HIV patients was A) **Working**: Able to perform usual work inside or outside the home. B) **Ambulatory**: Able to perform Activity of Daily Living (ADL), Not able to work. C) **Bedridden**: Not able to perform ADL [19].

### Ethical considerations

Ethical approval was obtained from Jimma University Institutional Review Board (IRB) with Ref. No. Phar/696/2013 and sent to the ART/TB follow-up care clinic. Verbal informed consent was obtained from the director of the ART/TB clinic and health professionals working at the clinic prior to patients’ card review after the purpose of the study was explained to them. Confidentially of the patients’ data was kept by using unique identification codes rather than patient names.

## Results

### Sociodemographic characteristics of the HIV-1 patients

One hundred fifty HIV-1 infected patients were included. Eighty-two (54.7%) of the patients were female. The median (IQR) age of the patients was 33.5 (25.7, 40.0) years. More than half (58.0%) of the patients were married and living in the urban area (53.3%). Seventy (46.7%) of the patients were unemployed, and the least were housewives (6.0%) and government employers (6.7%). Half of the patients had primary education and fewer patients had an educational level of tertiary and above (4.7%). Regarding the social drug use, 46.0% of the patients were a current/history of khat chewer and 38.0% alcohol drink (Table 1).

**Table 1:** Sociodemographic characteristics of the HIV-1 infected patients at ART clinic of JMC

Variables		Frequency	Percentage
Sex	Male	68	45.3
	Female	82	54.7
Age, median (IQR) years, 33.5 (25.7, 40.0)			
Marital status	Single	41	27.3
	Married	87	58.0
	Divorced	16	10.7
	Widowed	6	4.0
Residence	Urban	80	53.3
	Rural	70	46.7
Education level	Illiterate	43	28.7
	Primary	75	50.0
	Secondary	25	16.7
	Tertiary and above	7	4.7
Occupation	Unemployed	70	46.7
	Students	22	14.7
	Merchant	22	14.7
	Farmer	17	11.3
	Government	10	6.7
	House wife	9	6.0
Khat chewing		69	46.0
Alcohol drink		57	38.0
Smoking		47	31.3

*IQR* Interquartile range

### Clinical characteristics of HIV-1 infected patients

Two-third (66.0%) of the patients had WHO clinical stage I and II. One hundred ten (73.3%) of the patients had the functional status of ambulatory and 2.0% of the patients had bedridden functional status at baseline. The median (IQR) CD4 cells was 414.0 (210.0, 650.0) cells/μL. Fifty-nine (39.3%) of the patients had CD4 cells greater than 500 cells/μl and 22.7% of the patients had less than 200 cells/ μL. Viral load (VL) measurement was reported in 14 patients. The mean (SD) VL was 2051.6 ± 715.8 copies/μL. One patient had a VL of greater than 1000 copies/ml. The median (IQR) of the body mass index (BMI) of the patients was 20.2 (18.4, 22.9) kg/m^2^. Eighty-eight (58.7%) of the patients had normal BMI while 26.0% of patients had a BMI less than 18.5 kg/m^2^. Regarding the comorbid diseases, 26 (17.3%) of the patients had comorbid diseases. Twenty-six (17.3%) of the patients had a family history of TB (Table 2).

**Table 2:** Clinical characteristics of HIV-1 infected patients at ART clinic of JMC

Parameters		Frequency	Percentage
WHO stages	I	66	44.0
	II	33	22.0
	III	35	23.3
	IV	16	10.7
Functional status	Working	110	73.3
	Ambulatory	37	24.7
	Bed ridden	3	2.0
CD4 cells	Less than 200	34	22.7
	200–350	33	22.0
	350–500	24	16.0
	Greater than 500	59	39.3
BMI (kg/m^2^)	Less than 18.5	39	26.0
	18.5–24.9	88	58.7
	Greater than 25	23	15.3
Comorbid diseases		26	17.3
Family history of TB		26	17.3

*BMI* Body Mass Index, *WHO* World Health Organization, *CD4* Cluster of differentiation 4

### ART medications, Cotrimoxazole prophylaxis and isoniazid preventing therapy (IPT)

One-hundred twenty-two (81.3%) of the patients were taking Tenofovir + Lamivudine + Efavirenz (TDF + 3TC + EFV). Based on the pill count adherence measurement, 94.7% of patients had good adherence and 5.3% had poor ART adherence. One hundred thirty-six (90.7%) of the patients were received cotrimoxazole prophylaxis while 81.3% of the patients received IPT (Table 3).

**Table 3:** ART medications, Cotrimoxazole prophylaxis and Isoniazid preventing therapy (IPT) among HIV-1 infected patients at ART clinic of JMC

Medications		Frequency	Percentage
ART	TDF + 3TC + EFV	122	81.3
	AZT + 3TC + NVP	20	13.3
	TDF + 3TC + DTG	8	5.3
Adherence to ART	Good	142	94.7
	Poor	8	5.3
Cotrimoxazole prophylaxis		136	90.7
Isoniazid preventive therapy		122	81.3

*ART* Anti-retroviral, *TDF* Tenofovir, *3TC* Lamivudine, *EFV* Efavirenz, *AZT* Zidovudine, *NVP* Nevirapine, *DTG* Dolutegravir

### Prevalence of active TB infection

**Fig. 1:**
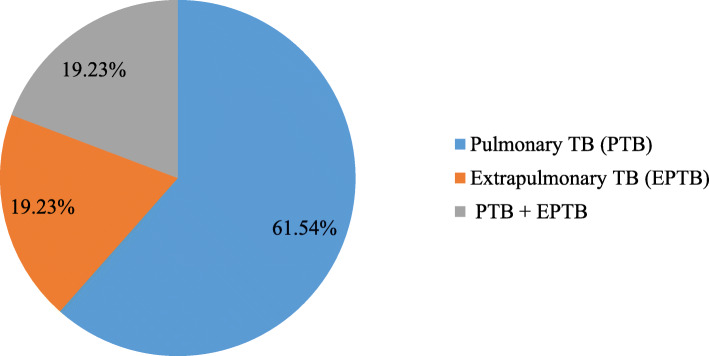
Pie chart of the types of TB infection among HIV-1 infected patients at ART clinic of JMC

Of the total of 150 HIV-1 infected patients, 17.3% of the patients had developed active TB infection. Nineteen (73.08%) of the TB patients had positive acid-fast bacilli (AFB). Similarly, the majority (61.54%) of the TB patients had a pulmonary TB infection (Fig. 1).

### Factors associated with the development of active TB infection among HIV-1 infected patients

Upon the univariate logistic regression analysis, having no formal education, family history of TB, WHO clinical stage III & IV, baseline CD4 cell count less than 350 cells/μL, ambulatory and bedridden functional status, and presence of other comorbid diseases significantly increased the risk of developing active TB infection. While, the use of isoniazid preventive therapy, cotrimoxazole prophylaxis, and having good adherence to ART medications significantly reduced the risk of active TB infection.

However, the multivariate logistic regression analysis showed, WHO clinical stage III and IV, the use of isoniazid preventive therapy, and having good adherence to the ART medications significantly associated with active TB infection. Patients in WHO clinical stage III and IV were more than 9 times more likely to develop TB infection (AOR: 9.67, 95% CI; 2.21–42.37), *p* = 0.003), compared to patients with WHO clinical stage I and II. However, the use of isoniazid preventive therapy reduced the risk of TB infection by 87.7% (AOR: 0.12, 95CI; 0.03–0.44, *p* = 0.001) compared to patients who did not use isoniazid preventive therapy. Similarly, HIV-infected patients who were assessed to have good adherence to ART medications were less likely to develop TB infection (AOR: 0.076, 95CI; 0.007–0.80, *p* = 0.032) (Table 4).

**Table 4:** Multivariate logistic regression analysis of factors associated with the development of active TB infection among HIV-1 infected patients at ART clinic of JMC

Variables		COR	Sig.	AOR	95 conf. Interval	Sig.
Education level	Illiterate	2.60	0.034	1.156	0.28–4.74	0.840
	At least primary (reference)	1				
Khat chewing	Yes	1.77	0.192	1.006	0.26–3.88	0.993
	No (reference)					
Family history of TB	Yes	8.54	< 0.001	2.286	0.502–10.41	0.285
	No (Reference)	1				
WHO clinical stage	III & IV	26.30	< 0.001	9.67	2.21–42.37	0.003
	I & II (reference)	1				
CD4 cell count/μl	< 350	9.66	< 0.001	0.456	0.058–3.57	0.455
	> 350 (reference)	1				
Functional status	Ambulatory and bed ridden	3.60	0.004	1.713	0.45–6.51	0.429
	Working (Reference)	1				
Comorbidities	Yes	4.22	0.003	2.441	0.57–10.43	0.228
	No (Reference)	1				
IPT	Yes	0.040	< 0.001	0.123	0.034–0.44	0.001
	No (reference)	1				
Cotrimoxazole prophylaxis	Yes	0.114	< 0.001	0.243	0.051–1.16	0.076
	No (Reference)	1				
ART adherence	Good	0.022	0.001	0.076	0.007–0.80	0.032
	Poor (Reference)	1				

*Keys*: *IPT* Isoniazid preventive therapy, *ART* Anti-retroviral therapy, *C/AOR* crude/adjusted odds ratio

## Discussion

HIV infection is the most powerful known risk factor predisposing for *Mycobacterium tuberculosis* infection and progression to active disease. The co-infections of TB and HIV pose particular diagnostic and therapeutic challenges and exert immense pressure on health care systems in African countries with large populations of co-infected individuals [20]. Therefore, understanding the predictors of active TB infection among HIV-infected patients is paramount to improve TB/HIV co-infected patients’ management. As a result, this retrospective cross-sectional study was conducted with the intention to determine the prevalence and factors associated with the development of TB infection among HIV-1 infected patients.

In this study, the number of HIV-1 infected female patients outweighs the number of male patients, and the median age for all patients was 33.5 years. And most of them are from urban areas. Several studies from Ethiopia reported similar findings. Debre Markos [13], Yirgalem [7], Gondar [11], Debre Tabor [14], Addis Ababa [21], Amhara region [9]. This is because one of the hotspots for HIV infection in Ethiopia was urban and being in middle age was also a risky group to acquire HIV infection [22]. Most of the HIV- infected patients in this study were in WHO clinical stage I and had a working functional class. Our finding is comparable with reports from Hawassa [10], Gondar [11], Debre Tabor [14], Debre Markos [13], and Gojjam [15]. Less than a quarter (23.0%) of the patients had CD4 cell counts of less than 200 cell/μL. Comparable findings were reported from Yirgalem [7] and Gojjam [15]. However, relatively higher findings were reported from Gondar [11] and Debre Tabor [14], and Amhara region [9]. The differences in the CD4 counts from the different studies could be due to the different sociodemographic of the patients and timing of presentation to the health facilities.

Our study showed the proportion of TB occurrence among the HIV-1 infected patients was 17.3%. This finding is supported by the previous Ethiopian studies from Debre Markos (16.9%) [13] and Hawassa (18.2%) [10]. However, studies from Afar (26.4%) [23] and Addis Ababa (25.8%) [24] reported a higher TB occurrence. Both of the studies were multicenter and employed relatively a higher sample size as compared to our study which is a unit center with a relatively lower sample size. This could be the possible explanation for the discrepancy. On the contrary, studies from Gondar (6%) [12] and (7.5%) [11], Debre Markos 12.55% [16], Arba Minch (7.2%) [8], and Tanzania (11%) [25] reported the proportion of TB occurrence among HIV-infected patients was lower compared to our study finding. This inconsistency could be due to the difference in the study design, patient characteristics, geography, and diagnostic modalities employed.

Concerning the type of TB infection, this study indicated 19.2% of the HIV-1 infected patients had extrapulmonary TB. Similar findings were reported in a meta-analysis from Sub-Saharan Africa [26] and a retrospective study from Ethiopia [16]. However, the finding of this study was relatively higher compared to studies by Fikadu et al. [10] and Alemu et al. [21], where 14.0 and 12.95% of the TB cases were extrapulmonary, respectively. In a study by Ahmed et al. [23], 76.47% of the HIV/AIDS patients had extrapulmonary TB. This difference might be due to the higher prevalence of active TB infection in a study by Ahmed et al. [23] compared to our study.

In the present study, patients in WHO clinical disease stages III and IV were more than 9 times more likely to develop TB infection compared to those patients with WHO clinical stages I and II. This finding is in line with reports of previous Ethiopian studies from Debre Markos [13], Afar [23], and Hawassa [10]. A study from Tanzania [25] had also reported a similar finding. This implies that HIV-infected patients with advanced WHO clinical stages (stage III and IV) are at higher risk for TB and need to take IPT as per guideline recommendations. In support of this, the present study revealed that the use of IPT reduced the risk of TB infection by 87.7% compared to patients who did not use it. This is in line with the WHO recommendations of IPT use to reduce the occurrence of TB in HIV-infected patients [27] and the TEMPRANO ANRS 12136 trial [28]. This finding is also supported by multiple studies [13, 16, 23, 25].

In this study, the other determinant for the occurrence of TB infection was adherence to ART medications. HIV-1 infected patients who were assessed to have good adherence to the ART medications were less likely to develop TB infection. Non-adherence might increase the viral load, which might increase patients’ risk for TB infection. The finding is in line with studies done in West Shewa Zone [29] and Debre Markos [16].

### Limitation

Although this study is up-to-date in the area in our setting, relatively the small sample size, being cross-sectional in the design, and retrospective in nature of this study was the major limitation. The absence of other clinical variables may also be a limitation.

## Conclusions

The prevalence of tuberculosis infection in this study was 17.3%. Advanced WHO clinical stages increased the risk of developing TB infection. However, the use of isoniazid preventive therapy and having a good adherence to ART medications reduced the risk of TB infection among HIV-1 infected patients. Thus, we recommend clinicians to early screen the HIV-infected patients for tuberculosis and initiation of isoniazid preventive therapy particularly for patients with advanced WHO clinical stage. Clinicians should also educate the patients about the importance of adherence to their medications. These measures counteract the progression of latent TB to active TB; which in turn decreases the spread of tuberculosis.

## Data Availability

All data generated or analyzed during the study are included in this published article.

## Abbreviations

TB: Tuberculosis
HIV-1: Human immune-deficiency virus-1
AIDS: Acquired immune-deficiency syndrome
PLWH: People living with HIV
WHO: World health organization
ART: Anti-retroviral therapy
AOR: Adjusted odds ratio
IQR: Interquartile range
SD: Standard deviation
CI: Confidence interval
IPT: Isoniazid preventive therapy
JMC: Jimma medical center
VL: Viral load
BMI: Body mass index
PTB: Pulmonary TB
EPTB: Extrapulmonary TB
AFB: Acid-fast bacilli

## References

[1] World Health Organization (WHO) (2018). Tuberculosis and HIV.

[2] Bruchfeld J, Correia-Neves M, Kallenius G (2015). Tuberculosis and HIV coinfection. Cold Spring Harb Perspect Med.

[3] CDC (2016). TB and HIV Coinfection.

[4] Swaminathan S (2016). Tuberculosis/HIV co-infection. Int J Infect Dis.

[5] Ministry of Health (2018). Guidelines for Management of TB, DR-TB and Leprosy in Ethiopia.

[6] Dalbo M, Tamiso A (2016). Incidence and predictors of tuberculosis among HIV/AIDS infected patients: a five-year retrospective follow-up study. Adv Infect Dis.

[7] Negussie A, Debalke D, Belachew T, Tadesse F (2018). Tuberculosis co-infection and its associated factors among people living with HIV/AIDS attending antiretroviral therapy clinic in southern Ethiopia: a facility based retrospective study. BMC Res Notes.

[8] Mama M, Manilal A, Tesfa H, Mohammed H, Erbo E (2018). Prevalence of pulmonary tuberculosis and associated factors among HIV positive patients attending antiretroviral therapy Clinic at Arba Minch General Hospital, Southern Ethiopia. Open Microbiol J.

[9] Mitku AA, Dessie ZG, Muluneh EK, Workie DL (2016). Prevalence and associated factors of TB/HIV co-infection among HIV infected patients in Amhara region, Ethiopia. Afr Health Sci.

[10] Fekadu S, Teshome W, Alemu G (2015). Prevalence and determinants of tuberculosis among HIV infected patients in South Ethiopia. J Infect Dev Ctries.

[11] Wondimeneh Y, Muluye D, Belyhun Y (2012). Prevalence of pulmonary tuberculosis and immunological profile of HIV co-infected patients in Northwest Ethiopia. BMC Res Notes.

[12] Alemayehu M, Gelaw B, Abate E, et al. Active tuberculosis case finding and detection of drug resistance among HIV-infected patients: A cross-sectional study in a TB endemic area, Gondar, Northwest Ethiopia. Int J Mycobacteriol. 2014;3(2):132-8. 10.1016/j.ijmyco.2014.02.004. 10.1016/j.ijmyco.2014.02.004 PMC5030108 26786335

[13] Temesgen B, Kibret GD, Alamirew NM, et al. Incidence and predictors of tuberculosis among HIV-positive adults on antiretroviral therapy at Debre Markos referral hospital, Northwest Ethiopia: a retrospective record review. BMC Public Health. 2019;19:1566. 10.1186/s12889-019-7912-9. 10.1186/s12889-019-7912-9 PMC6880633 31771552

[14] Kiros T, Dejen E, Tiruneh M, Tiruneh T, Eyayu T, Damtie S, Amogne K (2020). Magnitude and associated factors of pulmonary tuberculosis among hiv/aids patients attending antiretroviral therapy clinic at Debre Tabor specialized hospital, Northwest Ethiopia, 2019. HIV/AIDS Res Palliat Care.

[15] Belew H, Wubie M, Tizazu G, Bitew A, Birlew T (2020). Predictors of tuberculosis infection among adults visiting anti-retroviral treatment center at east and west Gojjam, northwest, Ethiopia, 2017. BMC Infect Dis.

[16] Aemro A, Jember A, Anlay DZ. Incidence and predictors of tuberculosis occurrence among adults on antiretroviral therapy at Debre Markos referral hospital, Northwest Ethiopia: retrospective follow-up study. BMC Infect Dis. 2020;20(1):245. Published 2020 Mar 26. 10.1186/s12879-020-04959-y 10.1186/s12879-020-04959-y PMC7098113 32216747

[17] Gudina EK, Gudissa FG. Prevalence of tuberculosis in HIV in Ethiopia in early HAART era: retrospective analysis. Pan Afr Med J. 2013;14:126. Published 2013 Apr 1. 10.11604/pamj.2013.14.126.1907. 10.11604/pamj.2013.14.126.1907 PMC3670201 23734271

[18] Shukla M, Agarwal M, Singh JV, Tripathi AK, Kumar A (2016). Nonadherence to antiretroviral therapy among people living with HIV / AIDS attending two Tertiary Care Hospitals in District of Northern India. Indian J Community Med.

[19] Thejus T, Jeeja M, Jayakrishnan T. The functional status of patients with AIDS attending antiretroviral treatment center. Indian J Palliat Care. 2009;15(1):57-60. 10.4103/0973-1075.53513. 10.4103/0973-1075.53513 PMC2886209 20606857

[20] Pawlowski A, Jansson M, Sköld M, Rottenberg ME, Källenius G. Tuberculosis and HIV co-infection. PLoS Pathog. 2012;8(2):e1002464. 10.1371/journal.ppat.1002464. 10.1371/journal.ppat.1002464 PMC3280977 22363214

[21] Alemu A, Yesuf A, Gebrehanna E, Zerihun B, Getu M, Worku T, Bitew ZW (2020). Incidence and predictors of extrapulmonary tuberculosis among people living with Human Immunodeficiency Virus in Addis Ababa, Ethiopia: a retrospective cohort study. PLoS One.

[22] Adal M (2019). Systematic review on HIV situation in Addis Ababa, Ethiopia. BMC Public Health.

[23] Ahmed A, Mekonnen D, Shiferaw AM, Belayneh F, Yenit MK (2018). Incidence and determinants of tuberculosis infection among adult patients with HIV attending HIV care in north-East Ethiopia: a retrospective cohort study. BMJ Open.

[24] Alemu A, Yesuf A, Zerihun B, Getu M, Worku T, Bitew ZW. Incidence and determinants of tuberculosis among HIV-positive individuals in Addis Ababa, Ethiopia: A retrospective cohort study. Int J Infect Dis. 2020;95:59-66. 10.1016/j.ijid.2020.02.053. 10.1016/j.ijid.2020.02.053 32126324

[25] Gunda DW, Maganga SC, Nkandala I, Kilonzo SB, Mpondo BC, Shao ER, Kalluvya SE (2018). Prevalence and risk factors of active TB among adult HIV patients receiving ART in Northwestern Tanzania: a retrospective cohort study. Can J Infect Dis Med Microbiol.

[26] Mohammed H, Assefa N, Mengistie B (2018). Prevalence of extrapulmonary tuberculosis among people living with HIV/AIDS in sub-saharan Africa: a systemic review and meta-analysis. HIV/AIDS Res Palliat Care.

[27] Date AA, Vitoria M, Granich R, Banda M, Fox MY, Gilks C (2010). Mise en oeuvre de la prophylaxie par le co-trimoxazole et du traitement préventif par l’isoniazide chez les personnes vivant avec le VIH. Bull World Health Org.

[28] Danel C, Moh R, TEMPRANO ANRS 12136 Study Group (2015). A trial of early Antiretrovirals and isoniazid preventive therapy in Africa. N Engl J Med.

[29] Nugus GG, Irena ME (2020). Determinants of active tuberculosis occurrences after ART initiation among adult HIV-positive clients in west Showa zone public hospitals, Ethiopia: a case-control study. Adv Public Heal.

